# Predictive Value of Endoscopic Observations and Biopsy After Neoadjuvant Chemoradiotherapy in Assessing the Pathologic Complete Response of Patients With Esophageal Squamous Cell Carcinoma

**DOI:** 10.3389/fonc.2022.859079

**Published:** 2022-05-11

**Authors:** Ali Taghizadeh Kermani, Raha Ghanbarzadeh, Mona Joudi Mashhad, Seyed Alireza Javadinia, Ali Emadi Torghabeh

**Affiliations:** ^1^ Cancer Research Center, Mashhad University of Medical Sciences, Mashhad, Iran; ^2^ Student Research Committee, Mashhad University of Medical Sciences, Mashhad, Iran; ^3^ Non-Communicable Diseases Research Center, Sabzevar University of Medical Sciences, Sabzevar, Iran

**Keywords:** Esophageal squamous cell carcinoma, endoscopic biopsy, neoadjuvant chemoradiotherapy, predictive value, neoadjuvant treatment

## Abstract

**Introduction:**

No standard method has been defined to evaluate the therapeutic response of esophageal cancer to neoadjuvant chemoradiotherapy (CRT). This study aimed to determine the predictive value of endoscopic evaluation and biopsy after CRT in predicting the complete pathological response to neoadjuvant CRT in patients with esophageal squamous cell carcinoma (SCC).

**Materials and Method:**

This prospective, descriptive study was conducted on patients with stage II and III esophageal SCC who could undergo esophagectomy. Patients underwent neoadjuvant CRT. Four to six weeks after the end of treatment, re-endoscopy was performed and a biopsy was taken in the presence of a tumor lesion. In the absence of a tumor lesion, the marked site of the esophagus was removed as a blind biopsy. Gastrologist observations during endoscopy and the result of the pathological examination of an endoscopic biopsy were recorded. The patient underwent esophagectomy. The pathology obtained from endoscopic biopsy was compared with the pathology response obtained from esophagectomy.

**Results:**

Sixty-nine patients were included in the study, of which 32 underwent esophagectomy. In an endoscopic examination after CRT, 28 patients had macroscopic tumor remnants and 4 patients did not. Pathological examination of the samples obtained from endoscopy showed no tumor remnants in 10 patients (31.3%), and in 22 patients (68.7%), living tumor remnants were seen in the biopsy specimen. Pathologic evaluation of the samples obtained by surgical resection showed that in 13 patients, there were no viable carcinomas in the esophagus or lymph nodes removed, and the rate of pathologic complete response was 40.6. Sensitivity, specificity, positive predictive, and negative predictive values of endoscopic observations were 94.7, 23, 64.2, and 75%, respectively. Preoperative biopsy sensitivity, specificity, positive predictive value (PPV), and negative predictive value (NPV) were 68.4, 30.7, 59, and 40%, respectively.

**Conclusion:**

Considering the negative and positive predictive values of endoscopic observations and biopsy after neoadjuvant CRT, it seems that these two methods alone are not suitable for assessing the pathologic complete response after neoadjuvant treatment.

## Introduction

Malignancies are one of the most common causes of death in developing and developed countries, accounting for a large portion of the annual health system expenditures in these countries. Meanwhile, esophageal cancer does not have a significant prevalence worldwide, but due to its regional prevalence pattern, it is still highly prevalent in areas such as the north and northeast of Iran. It is the second most common cancer in Iranian men and the third among Iranian women (1, 2). Despite advances in the diagnosis and treatment of esophageal cancer and the relative reduction in mortality due to them, this cancer is still considered one of the most lethal (3).

The standard treatment for esophageal cancer in patients with curative intent is esophagectomy. However, methods such as tumor removal by endoscopy, neoadjuvant, or definitive CRT are used depending on the condition of the patient. In most cases, staging is performed before starting treatment to select the appropriate treatment using endoscopic ultrasonography with CT scan. Neoadjuvant CRT in patients with operable esophageal cancer in stages IIB and III has significantly increased the survival of patients and is the recommended treatment (4, 5).

Although esophagectomy is a difficult operation with many complications, currently the only way to evaluate the response of an esophageal tumor to neoadjuvant treatment is to examine the samples from esophagectomy and lymphadenectomy (6), and a standard method for evaluating the therapeutic response of esophageal tumors to preoperative CRT in patients has not been defined before surgery. In the presence of a reliable method for evaluating the therapeutic response, this heavy surgery could be avoided. In numerous studies, various combinations of fluorodeoxy glucose positron emission tomography (FDG-PET) scan, computed tomography (CT) scan, endoscopy, and esophagography in cohorts of 60–280 patients have found a physician-assessed clinical response to have an accuracy of between 46 and 79% and an NPV of between 31 and 74% (7–9). From these, FDG-PET scan was more accurate and the reduction in FDG uptake in the tumor site was associated with pathologic complete response (10), but this method is expensive and has some limitations too. This study determined the predictive value of endoscopic evaluation and biopsy after DRT in predicting the complete pathological response to neoadjuvant CRT in patients with esophageal SCC.

## Materials and Methods

This prospective descriptive study was conducted in the radiation oncology department of Imam Reza Hospital and Omid Hospital, affiliated with Mashhad University of Medical Sciences during 2017 and 2018. Inclusion criteria included patients with stage II and III esophageal SCC whose disease was confirmed by endoscopic biopsy and tissue examination by a pathologist, and whose clinical condition (in terms of comorbidities) allowed esophagectomy. The exclusion criteria were the presence of distant metastasis, failure to complete the treatment protocol by patients, failure to perform esophagectomy for any reason after completing the course of CRT, and dissatisfaction with participating in the study. During primary endoscopy, the location of the tumor for future interventions was determined using anatomical criteria. Patients underwent thoracic and abdominal CT scans to stage the disease. Following written consent, patients received weekly carboplatin (AUC = 2) and paclitaxel (50 mg/m^2^) chemotherapy for five weeks, followed by 28 sessions of radiotherapy with a final dose of 5,040 centigray (cGY) and 180 cGY/fraction. Four to six weeks after the end of CRT, endoscopy was performed again and a biopsy was taken from tumor. In the absence of a tumor lesion, the esophageal marked site was removed as a blind biopsy. Gastrologist observations and the result of the pathological examination of endoscopic biopsy were recorded. Then the patient was referred for surgery, and after esophagectomy, the sample was sent for pathology, and finally, the pathology results obtained from endoscopic biopsy were compared with the pathology results obtained from esophagectomy. Data were entered into the SPSS 21 software and descriptive statistics such as mean, standard deviation, frequency, percentage, sensitivity and specificity, positive and negative predictive value, positive and negative probability, and preoperative biopsy accuracy in predicting postoperative pathology response were reported.

## Results

Sixty-nine patients were enrolled in the study, of whom 32 underwent esophagectomy after neoadjuvant CRT. In the 32 patients who underwent surgery, the median age was 65.5 years. Other patient characteristics are listed in **Table 1**.

**Table 1:** Background characteristics of the subjects at the beginning of the study.

Variable	Percent (Number)
	
Gender	
Male	53 (17)
Female	47 (15)
Tumor site	
Middle esophagus	47 (15)
Lower esophagus	53 (17)
Tumor grade	
I	25 (8)
II	62.5 (25)
III	12.5 (4)
Duration of CRT	
5 Weeks	43.8 (14)
6 Weeks	40.6 (13)
7 weeks	15.6 (5)
Radiation dose	
50 or 50.4 Gy	59.4 (19)
Higher dose	40.6 (13)

CRT, Chemoradiotherapy; Gy,Gray.

Endoscopic observations after neoadjuvant CRT showed macroscopic remnants of the tumor in 28 patients (87.5%), and 4 patients (12.5%) had no macroscopic remnants. Pathological examination of endoscopic biopsy after neoadjuvant treatment showed that in 10 patients (31.3%), there was no viable tumor. In 22 patients (68.7%), tumor remnants were seen in the biopsy specimen. Evaluation of the samples obtained from surgical resection showed that in 13 patients, there were no tumor remains in the esophagus or lymph nodes removed, so the rate of pathologic complete response was 40.6. Of the 19 patients with tumor remnants, 13 (40.6%) reported T + (three were yT1, seven were yT2, and three were yT3) and six were yT0 N^+^. Also, in the lymph node evaluation, the results showed that 10 patients (31.3%) had yN1.

The results of the diagnostic accuracy of endoscopic observations after CRT showed that the frequency of true positive, false positive, true negative, and false negative macroscopic findings in this study were 18, 10, 3, and 1, respectively. **Table 2** shows the frequency of positive and negative material from macroscopic findings in preoperative endoscopy in predicting the true pathological response of the tumor. As shown in the table, the sensitivity and specificity of endoscopic macroscopic findings after CRT in predicting tumor pathology response after esophagectomy are 94.7 and 23%, respectively. Also, the PPV (cancer remaining in esophagectomy sample) and NPV (no cancer remaining in esophagectomy sample) in predicting pathologic complete response are 64.2 and 75%, respectively. Overall, the accuracy of endoscopic macroscopic findings after CRT in predicting the pathologic complete response after esophagectomy is 65.6%.

**Table 2:** Frequency of true positive, false positive, true negative, and false negative macroscopic findings during endoscopy.

Surgical results			
	Positive	Negative	Total
**Positive**	18	10	28
**Negative**	1	3	4
**Total**	19	13	32

### Macroscopic Findings

Examination of the diagnostic accuracy of biopsy during endoscopy after CRT showed that the frequency of true positive, false positive, true negative, and false negative microscopic findings in biopsy specimens were 13, 9, 4, and 6, respectively (**Table 3**). The sensitivity and specificity of endoscopic biopsy findings after CRT in predicting pathological response after esophagectomy were 68.4 and 30.7%, respectively. Also, the positive and negative predictive values of this method in predicting the pathologic complete response are 59 and 40%, respectively. Overall, the accuracy of microscopic findings from endoscopic biopsy after CRT in predicting the pathologic complete response after esophagectomy is 53.1%.

**Table 3:** Frequency of true positive, false positive, true negative, and false negative of microscopic.

Microscopic findings Surgical results	Positive	Negative	Total
**Positive**	13	9	22
**Negative**	6	4	10
**Total**	19	13	32

## Discussion

In this study, we sought to answer the question of whether it is possible to rely on endoscopic and biopsy findings after neoadjuvant CRT to ensure the response of esophageal SCC, follow up the patients based on these findings, and not recommend surgery. Numerous studies have been conducted in this field. For example, in a study by Yang et al., 183 patients with locally advanced esophageal and gastroesophageal junction carcinoma who underwent neoadjuvant therapy and then surgery were retrospectively evaluated. Of these patients, 65 cases underwent esophageal biopsy after CRT, which reported remaining cancer cells in the biopsy specimen of 20% (13 patients), and in 52 patients, no remnants of cancer cells were reported. Examining the relationship between esophageal biopsy results after CRT and residual tumor status in esophagectomy specimens, the results showed that there was no significant difference in cancer residual status in esophagectomy specimens between patients with positive biopsy and patients with negative biopsy. The PPV of esophageal biopsy after CRT was 92.3% and the NPV was 23.1%. The sensitivity of endoscopic biopsy after neoadjuvant treatment was 23.1% and its specificity was 92.3%. This study concluded that endoscopic biopsy after neoadjuvant therapy is a specific but not sensitive method for predicting post-esophagectomy cancer remnants (11).

Schneider et al. studied the response of esophageal cancer to neoadjuvant CRT by endoscopy, biopsy, and endoscopic ultrasonography. Ninety-one patients were evaluated. The results of re-biopsy evaluation after neoadjuvant CRT showed that 69.7% had no evidence of tumor cells (negative result) and 30.3% had tumor cells (positive result) in at least one sample. The evaluation of response by re-biopsy had a sensitivity of 36.4%, specificity of 100%, PPV of 100%, NPV of 23.9%, and accuracy of 47% in predicting histopathological response. This study concludes that the use of endoscopy and re-biopsy is not accurate enough to predict the histopathological regression after neoadjuvant CRT (12).

In their study, Sarkaria et al. examined 443 patients with esophageal cancer from 1996 to 2007. These patients received neoadjuvant CRT and then underwent esophagectomy. From these, 221 patients underwent endoscopy and 156 patients underwent endoscopic biopsy after neoadjuvant treatment. Of the 156 patients who underwent biopsy, 75.6% were negative and 24.4% were positive for malignancy. Patients who had a positive biopsy result after neoadjuvant treatment had more macroscopic remnants of endoscopy than patients with a negative biopsy. The results of this study showed that patients with a negative biopsy result were more likely to have a pathologic complete response. The sensitivity of endoscopic biopsy after CRT in predicting the pathologic complete response was 30.8% and its specificity was 94.9%. The positive and negative predictive values of this method were 94.7 and 31.4%, respectively. This study concludes that a negative endoscopic biopsy is not a useful predictor of the pathologic complete response following CRT, lymph node status, and survival (13).

In a study by Miyata et al. on the prognostic value of endoscopic biopsy findings after induction therapy with or without surgery for esophageal cancer, 169 patients who underwent endoscopic biopsy following induction CRT were evaluated. Of these, 123 underwent neoadjuvant CRT and then surgery. The study of the relationship between endoscopic biopsy after neoadjuvant CRT with pathologic outcome and survival showed that the biopsy result was negative in 50% of cases (61 out of 123 patients). Sensitivity, specificity, PPV, and NPV of endoscopic biopsy following neoadjuvant CRT in predicting pathologic complete response were 58.9, 78.6, 90.3, and 36.1%, respectively (14).

The findings of this study showed that the accuracy of endoscopic and biopsy findings after CRT in examining the tumor response to neoadjuvant treatment was more than 50%. Positive results of these methods on tumor remnants are more reliable than negative results. Because the response rate to neoadjuvant therapy in the involved lymph nodes in these evaluations cannot be assessed, the sensitivity, specificity, positive, and negative predictive values of these studies alone are unreliable and there is a need for additional studies. **Table 4** compares the results of this study with those mentioned in the study of endoscopic biopsy after neoadjuvant treatment. This study is the first evaluation in northeastern Iran with the aim of finding a diagnostic method to predict the response of esophageal cancer to neoadjuvant therapies other than esophagectomy. This study is limited to patients with esophageal SCC, so generalizing its results to patients with esophageal adenocarcinoma is limited. Another limitation of this study is the small sample size, the main reason being the lack of referral of patients for esophagectomy after CRT.

**Table 4:** Comparison of microscopic findings value of endoscopic biopsy after CRT in several studies.

Study	Patient Number	PPV (%)	NPV (%)	Sensitivity (%)	Specificity (%)
Yang	65	92.3	23.1	23.1	92.3
Schneider	91	100	23.9	36.4	100
Sarkaria	156	94.7	31.4	30.8	94.9
Miyata	123	90.3	36.1	58.9	78.6
Our Study	32	59	40	68.4	30.7

## Conclusion

The NPV of endoscopic observations and biopsy after neoadjuvant CRT is 75 and 40%, respectively, and the PPV of these two methods is about 64 and 59%, respectively. So, these two methods alone are inappropriate tools for assessing the pathologic complete response after neoadjuvant treatment of esophageal SCC.

## Data Availability Statement

The raw data supporting the conclusions of this article will be made available by the authors, without undue reservation.

## Ethics Statement

The studies involving human participants were reviewed and approved by the Mashhad University of Medical Sciences. The patients/participants provided their written informed consent to participate in this study.

## Author Contributions

Conceptualization: ATK. Data curation: SJ and RG. Funding acquisition: ATK. Investigation: RG. Methodology: AET. Project administration: ATK. Resources: ATK. Software: SJ. Supervision: ATK. Validation: AET. Visualization: MJM. Roles/Writing original draft: SJ and AET. Writing—review & editing: ATK and AET. All authors listed have made a substantial, direct, and intellectual contribution to the work and approved it for publication.

## Funding

This research was fully funded by Mashhad University of Medical Sciences [grant number: 941476].

## Conflict of Interest

The authors declare that the research was conducted in the absence of any commercial or financial relationships that could be construed as a potential conflict of interest.

## Publisher’s Note

All claims expressed in this article are solely those of the authors and do not necessarily represent those of their affiliated organizations, or those of the publisher, the editors and the reviewers. Any product that may be evaluated in this article, or claim that may be made by its manufacturer, is not guaranteed or endorsed by the publisher.
